# Case Report: Takotsubo cardiomyopathy in a postoperative patient without cardiological disease

**DOI:** 10.12688/f1000research.122298.1

**Published:** 2022-06-06

**Authors:** Luis Coaguila-Cusicanqui, Vanessa Castillo-Atoche, Roberto Montalvo-Suyon, Yuriko Cavero-Reyes, Virgilio E. Failoc-Rojas

**Affiliations:** 1 Hospital Regional Lambayeque, Lambayeque, 14013, Peru; 2 Universidad Privada Norbert Wiener, Lima, 15079, Peru; 3 Instituto de Evaluación de Tecnologías en Salud e Investigación, EsSalud, Lima, 15079, Peru

**Keywords:** Takotsubo cardiomyopathy, electrocardiography, Peru

## Abstract

**Background:** Takotsubo cardiomyopathy (TC) is characterized by a clinical presentation that mimics acute coronary syndrome but is reversible. Alterations of Takotsubo in patients without previous heart disease remain a challenge for diagnosis.

**Case report:** We present a case of an 80-year-old patient from Peru. The patient underwent surgery, with the diagnosis of Chilaiditi’s syndrome. One day after surgery, she presented with dyspnea, tachycardia, and electrocardiographic changes. The diagnosis of Takotsubo syndrome with cardiogenic shock and renal failure on hemodialysis was made. She was hospitalized in the Intensive Care Unit and was managed with vasopressors and nitroglycerin. There was no cardiac lesion in the cineangiogram or occlusion of arteries. The patient was extubated and received daily dialysis until discharge.

**Conclusions**: Takotsubo is an emotional, non-cardiac, or post-traumatic stressful event that triggers myocardial injury with segmental anomalous, the possible etiology of which is the release of an endothelial neurotransmitter caused by stress. Emergency physicians should be aware of this as even patients without previous cardiac pathologies when exposed to stressors (such as surgeries) develop emergency symptomatology similar to myocardial infarction. Thus, emergency physicians should identify any cardiac abnormalities after a stressor, as well as be prepared for the diagnosis of TC.

## Introduction

Takotsubo cardiomyopathy (TC), also known as stress cardiomyopathy, apical-ballooning syndrome or broken heart syndrome, is characterized by a clinical presentation that mimics acute coronary syndrome (ACS). ^1^ The estimated prevalence of TC is between 2% and 3% in patients with suspected ACS, but it can be higher reaching up to 10% in women. ^1^^,^^2^

TC presents a transient regional systolic dysfunction of the left ventricle that is extended beyond the coronary artery supplying area and seems to follow the anatomic cardiac sympathetic innervation. ^2^^,^^3^ This mimics an ACS (myocardial infarction with ST-segment elevation) or unstable angina. ^4^

The most frequent symptoms are chest pain, dyspnea, or syncope (75.9%, 46.9%, and 7.7%, respectively) according to the International Takotsubo Registry. ^5^ There are still uncertainties concerning its pathophysiology, diagnosis, and treatment. ^6^

This case report is about an elderly woman who was admitted to hospital for surgery, but in the post-operative phase, she presented with a complication similar to ACS, which was later diagnosed as TC. Here, we report the clinical symptoms and how the patient’s condition was improved.

## Case report

We present a case of an unemployed 80-year-old female patient from Cajamarca, Peru with a history of arterial hypertension and Type 2 diabetes mellitus for the last 10 years by which she has followed irregular treatment. She was admitted for an emergency to a hospital in Lambayeque, in the North West of Peru. On admission, the patient complained of stabbing abdominal pain in the right hemiabdomen, nausea, and constant vomiting after suffering a fall from approximately one meter. Once in the emergency department, she was evaluated by the surgery service that made the diagnosis of closed abdominal trauma and Chilaiditi’s syndrome and she was taken to the operating room to undergo surgery. A day after admission for surgery, she was taken to the recovery room. There, she started presenting with moderate dyspnea and tachycardia. After performing a Holter electrocardiogram (ECG), sinus rhythm, paroxysmal atrial fibrillation, and infrequent ventricular extrasystoles without abnormalities in the ST-segment were found. Echocardiography was also carried out, which revealed that the patient presented 75% preserved left ventricular ejection fraction (LVEF) with preserved motility, no hypertrophy or dilation of cavities, and Type 1 diastolic dysfunction. During the first five days after admission for an emergency, the patient continued presenting dyspnea, which was aggravating until it became severe with added oliguria. This was a diagnostic challenge because the description on the ECG suggested ACS or some other cardiac pathology (which had previously been ruled out). Seven days after admission for emergency and three days after surgery, she was admitted to the Intensive Care Unit (ICU) with a thrombolysis in myocardial infarction (TIMI) risk score of nine (probability of mortality of 35.9% over 30 days). In the ICU, the patient started with severe respiratory insufficiency, marked hypotension, and anuria, requiring intubation and ventilation support, hemodynamic support with vasopressor therapy (norepinephrine by continuous intravenous infusion at a dose of 0.12 and 004 μg/kg/min each day), and dialysis support.

**Figure 1.**
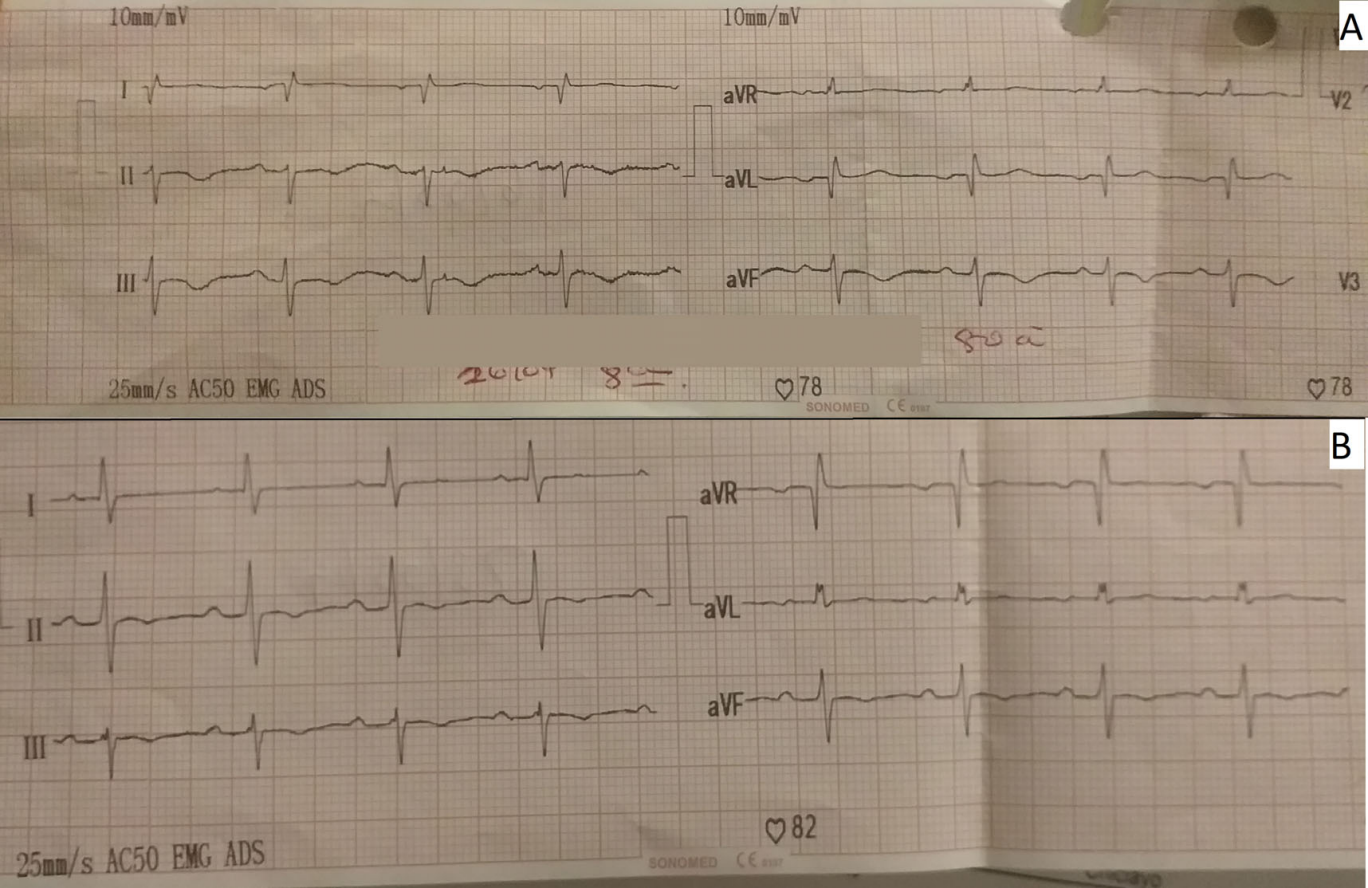
A: Negative T waves, in II, III and aVF. B: Nine days after treatment, with resolution of negative T waves. aVF, augmented vector foot; aVR, augmented vector right; aVL, augmented vector left.

**Figure 2.**
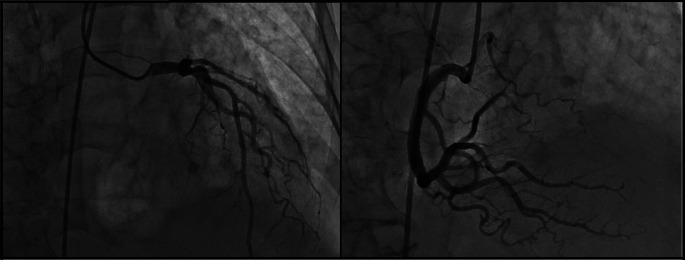
Cardiac characterization of the patient showing permeable coronary arteries with apical dyskinesia.

The day after she was admitted to the ICU (post-operative day four), the patient presented with atrial fibrillation with high ventricular response; then, amiodarone infusion was administered reverting the dysrhythmia. At the moment of the dysrhythmia, serial ECG was performed in which marked negative T-waves in II, III, and augmented vector foot (aVF) leads, and all precordial leads were observed ( Figure 1). Neither ST-segment disorder nor pathologic Q-waves were present. For this reason, the cardiology department performed echocardiography for the second time (post-operative day eight) where 44% LVEF systolic dysfunction, dilated left ventricle, mild mitral insufficiency, and severe aortic insufficiency with marked apical dyskinesia and contraction of basal segments were evidenced. In the cineangiogram, neither lesions nor occlusion were found in the trunk of the left coronary artery, anterior descending artery, circumflex artery or right coronary artery ( Figure 2).

Suspecting of ACS, anti-ischemic therapy was begun with digoxin 0.125 mg per day and cardiac enzymes were required, which resulted in troponin T elevation (0.35 ng/ml) and troponin I (0.55 ng/ml). Considering these results, coronarography was performed where no coronary lesions with natriuretic peptide (pro B-type natriuretic peptide (ProBNP)) 35,000 pg/ml (normal values: 0-222 pg/ml) were evidenced.

The diagnosis of Takotsubo syndrome with cardiogenic shock and renal failure on hemodialysis was made. An added septic process was ruled out with cultures and procalcitonin, which were negative. To complete the diagnostic study, abdominal transmission electron microscopy (TEM) and renal echography were conducted, ruling out a neoplastic process, pheochromocytoma or any other spreading process.

A total of 10 days after admission to the ICU (post-operative day 13), the patient ceased presenting shock; so, vasopressor infusion was discontinued (norepinephrine by continuous intravenous infusion at a dose of 0.12 and 004 μg/kg/min, which she received for two days). It should be noted that dobutamine by continuous intravenous infusion was also administered, but only for 24 hours (a dose of 5 μg/kg/min); then it was discontinued because the patient started presenting hypotension and tachyarrhythmia.

A total of 13 days after admission to the ICU (post-operative day 16), continuous intravenous infusion of nitroglycerin was given at a dose of 20–40 mcg/min, which was administered for 24 hours and control echocardiography was performed during the infusion of nitroglycerin, which showed that the patient was not presenting with systolic dysfunction (LVEF: 65%), motility was preserved with Type 1 diastolic dysfunction, with mild aortic and mitral insufficiency, and no apical dyskinesia. On echocardiographic findings, up to the date of reversion of the clinical picture of marked systolic dysfunction, negative T-waves persisted without evidence of ST-disorder or pathological Q-waves.

Infusion of nitroglycerin was discontinued by evidence of clinical improvement. Therefore, the patient continued treatment with atorvastatin (sublingual, 40 mg/day), enoxaparin (subcutaneous injection, 40 mg/day), digoxin (intravenous, 0.125 mg/day), and daily dialysis support.

The patient was removed from mechanical ventilation, being extubated 15 days after hospitalization in the ICU (post-operative day 18). She continued with non-invasive ventilator assistance for a further three days. Then, she was continued with low flow oxygen therapy with *F*IO _2_ 0.30 *via* nasal cannula. After that, the patient was discharged from the Internal Medicine service with daily dialysis support because of persistent anuria. Before discharge, pheochromocytoma, intracranial involvement, previous ischemic heart disease and severe organic valvular heart disease could be excluded. The patient progressed favorably until discharge.

At three months of follow-up, the patient did not present any hospital readmission, preserved systolic function (LVEF: 62%) and no other cardiac abnormalities, however, the patient eventually reported precordial pain without becoming disabling.

## Discussion

TC implies a diagnostic challenge because the initial clinical picture may be difficult to differentiate from ACS. TC is not uncommon but there are not many reports in Peru, perhaps due to the lack of adequate clinical suspicion.

It has been observed that TC affects women (85–90%) aged between 65 and 70 years old more often than men, ^5^^,^^6^ which is similar to what was observed in the present clinical case. This could be explained by the fact that estrogens regulate the myocardial sympathetic tone and vascularization in women of reproductive age. This sympatholytic effect of estrogens is lost after menopause, leading to an effect in the abnormality of the contraction of the left ventricle of TC. ^7^ Other causes have been related to the higher frequency of emotional or physical stress. ^1^ We should also consider that TC can also appear in young women, children, and newborns. ^3^

The diagnosis was mainly made by addressing one of the four criteria of Mayo Clinic for the diagnosis of TC: 1) mid and apical dyskinesia of the left ventricular segments with regional wall motion abnormalities; 2) absence of obstructive coronary artery disease; 3) appearance of new ECG abnormalities (T-wave inversion); and 4) absence of pheochromocytoma or myocarditis. ^8^ That is why the diagnostic suspicion and the search for the disease allowed us to rule out other possible causes of cardiomyopathy and ACS. It is recommended to consider this differential diagnosis in patients with ACS.

The etiology of Takotsubo is still uncertain but may be associated with catecholamine elevations during times of emotional or physical stress, and we believe that a post-surgical catecholamine overload and a brain-heart connection hypothesis may have caused the Takotsubo syndrome in this case report, without the need for prior cardiac pathology. ^9^^,^^10^

Elevated troponin and natriuretic peptide (proBNP) levels were found in this case. These laboratory findings are frequent in patients with TC reported in international registries, ^5^ which is why it is considered as a diagnostic criterion.

The patient reported with TC was treated with norepinephrine and dobutamine at standard doses; however, thinking of a cardiogenic problem, may induce additional catecholaminergic stress, so TC may be precipitated by exposure to these two drugs. ^6^^,^^7^ The pathogenic role of catecholamines is explained because high levels of catecholamines have been observed in patients with TC as well as an inflection of the left ventricle. ^11^ Catecholamines can produce induced microvascular spasm or cardiac toxicity. ^6^

Cardiac complications are common in TC. Despite the absence of lesions in cineangiogram, complications like acute cardiac insufficiency (12–45%), atrial fibrillation (7–17%), cardiogenic shock (17–30%), and dysrhythmia (5–15%) ^5^^,^^8^^,^^12^ have been reported. Patients with TC have a high risk of developing atrial fibrillation, ranging from 7.75–17.57%. ^11^^,^^13^ In this case report, atrial fibrillation, ventricular extrasystoles, dysrhythmia, ST-segment abnormalities, and aorta and mitral insufficiency are registered. It is important to discuss that atrial fibrillation does not increase in-hospital mortality, but it can lead to higher levels of comorbidities such as ventricular dysrhythmias, longer hospital stays, and the development of cardiac arrest. ^11^^,^^14^

The prognosis is generally favorable because in-hospital mortality ranges from 1–8%. ^2^ In this case, the patient was discharged from the hospital with clinical improvement. It has been observed that mortality rates of TC are higher in male patients than in female ones (8.4% *vs.* 3.6%, respectively; p<0.001). ^14^

This case explains that stress cardiomyopathy has in-hospital mortality of up to 5%. Most deaths occur among patients that develop unstable manifestations, including cardiac arrest or cardiogenic shock. The recovery of the left ventricular contraction is gradual, generally from 1 to 2 weeks although it can be fast (within 48 hours) or late (up to 6 weeks).

One important problem to solve is the need to determine the risk factors and the pathophysiological mechanisms in this disease, as well as the physiology of the patient already recovered from TC to determine, with certainty, the specific care these patients require in the long term. It is also important to conduct more research on cardiac and endocrine functions to find out why this disease mostly affects women. Higher methodological quality studies should be carried out to determine the best therapeutic option for these patients.

It is proposed that when faced with an unexpected and severe stress response, the autonomic nervous system synthesizes sympathetic neuronal exits and adrenomedullary hormones. The epinephrine released from the adrenal medulla and the norepinephrine from the cardiac and extracardiac sympathetic nerves reach the adrenoceptors in the blood vessels and the heart. ^1^ In this case report our patient did not have previous cardiac complications, so this support would be based on the release of catecholamines due to stress that has an expression in the cardiac receptors.

The frequency of TC in patients with ACS is often underestimated since there is not yet full knowledge of this disease, so it is recommended that physicians also consider the differential diagnosis in a patient admitted to hospital with ACS and evaluate the electrocardiogram and early invasive coronary angiography.

## Conclusions

TC is a relatively benign and reversible disease, but as it is reported in this study, to reach the diagnosis and indicate appropriate treatment is highly complex. It is frequent in women and can occur as a complication of pathology or stress. Takotsubo is an emotional, non-cardiac, or post-traumatic stressful event that triggers myocardial injury with segmental anomalous, the possible etiology of which is an endothelial neurotransmitter caused by stress.

## Data availability

### Underlying data

All data underlying the results are available as part of the article and no additional source data are required.

## Consent

Written informed consent for publication of their clinical details and clinical images was obtained from the patient.
