# Supplementary material for: Case Report: Takotsubo syndrome in a postoperative patient without cardiological disease
Source: F1000Res. 2022 Nov 14;11:616. Originally published 2022 Jun 6. [Version 2] doi: 10.12688/f1000research.122298.2 (PMC11470228; doi:10.12688/f1000research.122298.2)
Supplement: Peer review history

# Reviewer report: Reviewer response for version 2

**DOI:** 10.5256/f1000research.140629.r326998

**Published:** 2024-10-10

**Authors:** Hawani Sasmaya Prameswari

**Affiliations:** 1 Heart Failure Division, Cardiology and Vascular Medicine, Hasan Sadikin General Hospital, Bandung, Jawa Barat, Indonesia; 2 Department of Cardiology and Vascular Medicine, Faculty of Medicine, Padjadjaran University, Bandung, Indonesia

This is an interesting case and authors describe the challenge in diagnosing TS in this case. However, the case report lack sufficient details regarding the diagnostic tests, particularly the echocardiography, as there are no videos or images illustrating the typical findings associated with TS. Furthermore, the ECG doesn't correlate with the case description; it mentions T-waves inversions during dysrhythmia, yet the ECG figure displaying T-wave inversions shows Sinus Rhythm (SR) rather than AF. 

 The authors should consider including echo images or videos in the report. Additionally, given that the patient presented with AF at an older age and exhibited no structural abnormalities on the echo, it would be prudent to inquire about thyroid function evaluation. 

 To enhance the accuracy of diagnosing TS, CMRI would be important after the patient has stabilized; however this was not performed in this case. The authors may provide further explanation regarding this decision not to proceed to CMRI in this case. 

 The discussion section is insufficient in explaining why TS occurred in this patient. It should specifically address whether it was relate to specific surgical procedures, such abdominal operations, or the type of anesthesia used or pain management post operatively that may have contributed to the development of TS. Additionally to  relationship of massive cathecolamin exposure as it is not adequately explained and explored in this case. 

 Finally, the authors should emphasize more in the discussion section the lessons learned that differentiate this case from other reported instances of TS.

Are enough details provided of any physical examination and diagnostic tests, treatment given and outcomes?

Partly

Is the case presented with sufficient detail to be useful for other practitioners?

Partly

Is sufficient discussion included of the importance of the findings and their relevance to future understanding of disease processes, diagnosis or treatment?

Partly

Is the background of the case’s history and progression described in sufficient detail?

Yes

Reviewer Expertise:

My area(s) of research are heart failure, advanced heart failure, cardiomyopathy, myocarditis, and pulmonary hypertension.

I confirm that I have read this submission and believe that I have an appropriate level of expertise to confirm that it is of an acceptable scientific standard, however I have significant reservations, as outlined above.

# Reviewer report: Reviewer response for version 2

**DOI:** 10.5256/f1000research.140629.r155720

**Published:** 2022-11-16

**Authors:** John D. Horowitz

**Affiliations:** 1 Freemasons Centre for Male Health and Wellbeing, South Australian Health and Medical Research Institute, School of Medicine, The University of Adelaide, Adelaide, SA, Australia

The authors have not revised the article to a satisfactory standard based on my previous report. As such I cannot recommend this article for indexing. My comments read below:

1. The critically important aspect of this woman’s case was the poor treatment of hypotension, to an extent whereby she needed dialysis. Not much is said about the cause of the hypotension, but as I indicated it is likely to be induced by inappropriate vasodilatation due to excessive (not impaired) responsiveness to nitric oxide, as well as inflammatory vascular leakage, and should certainly have been treated with volume expansion rather than catecholamines and nitroglycerine.
2. The idea that TTS is “transient” is mentioned 3 times in the revised text, despite extensive evidence that recovery is at best slow, that it is often incomplete, and that there is a substantial risk of both recurrence and of long-term mortality.
3. The authors do not appear to have properly researched the pathogenesis of TTS, simply saying that it is “not understood”. However, there are data on biased signalling at beta-2 adrenoceptors, induction of nitrosative stress, and of myocardial energetic impairment, none of these quoted.
4. The patient did not receive long-term ACE inhibitors, despite evidence that these improve outlook long-term, nor is this mentioned.
5. Nothing is said about the utility of cardiac MRI for establishing the diagnosis positively, rather than leaving it as a diagnosis of exclusion.
6. Nothing is said about the association with cancers.
7. The infusion rate of nitroglycerine was inappropriately high and would have resulted in loss of nitroglycerine effect rapidly ^1^. Perhaps a good thing.
8. The patient follow-up does not indicate long-term monitoring of resolution of NTproBNP levels, nor of normalisation of global longitudinal strain patterns on echocardiography, the normal gold standards.

Are enough details provided of any physical examination and diagnostic tests, treatment given and outcomes?

No

Is the case presented with sufficient detail to be useful for other practitioners?

No

Is sufficient discussion included of the importance of the findings and their relevance to future understanding of disease processes, diagnosis or treatment?

No

Is the background of the case’s history and progression described in sufficient detail?

Partly

Reviewer Expertise:

NA

I confirm that I have read this submission and believe that I have an appropriate level of expertise to state that I do not consider it to be of an acceptable scientific standard, for reasons outlined above.

## References

[1] : Stable angina and acute coronary syndromes are associated with nitric oxide resistance in platelets. *Journal of the American College of Cardiology* .2001;37(7) : 10.1016/S0735-1097(01)01238-4 1851-1857 10.1016/S0735-1097(01)01238-4 11401122

# Reviewer report: Reviewer response for version 1

**DOI:** 10.5256/f1000research.134270.r144262

**Published:** 2022-07-25

**Authors:** Takeshi Nishimura

**Affiliations:** 1 Department of Emergency and Critical Care Medicine, Hyogo Emergency Medical Center, Kobe, Japan

This report "Case Report: Takotsubo cardiomyopathy in a postoperative patient without cardiological disease" is well written, and this reviewer understands what the authors tried to review with their unique case.

 Points of review:

1. Describe the findings of the laparotomy. Chilaiditi's syndrome does not require a surgical treatment.
2. Add the information of cardiac enzyme (CK-MB).
3. Nitroglycerine is not appropriate to treat TC. Show the reason why authors applied this treatment.
4. Discussion is incoherent, each paragraph should include the topics authors would like to emphasize. The mixing of short and long paragraphs makes following the right discussion points difficult. 

Authors just addressed the knowledge which is already known in previous reports. Readers would like to know academic aspects from this case. To improve the quality of this case report, this reviewer recommends authors to reconsider the concept of this case.

 Also, I recommend the manuscript be proofread.

Are enough details provided of any physical examination and diagnostic tests, treatment given and outcomes?

Yes

Is the case presented with sufficient detail to be useful for other practitioners?

Yes

Is sufficient discussion included of the importance of the findings and their relevance to future understanding of disease processes, diagnosis or treatment?

No

Is the background of the case’s history and progression described in sufficient detail?

Yes

Reviewer Expertise:

Emergency medicine, Trauma, Intensive care

I confirm that I have read this submission and believe that I have an appropriate level of expertise to confirm that it is of an acceptable scientific standard, however I have significant reservations, as outlined above.

# Response

**Published:** 2022-11-01

**Authors:** Virgilio E Failoc-Rojas

**Affiliations:** Unidad de Investigación para la Generación y Síntesis de Evidencias en Salud, Universidad San Ignacio de Loyola, Lima, Perú, Peru

**Response to Reviewer 3**

1. Describe the findings of the laparotomy. Chilaiditi's syndrome does not require a surgical treatment.

***Response: Thank you. We have described the findings of laparotomy. Indeed, Chilaiditi's syndrome generally has conservative management. However, the main diagnosis was closed abdominal trauma, which did require immediate surgery.***

1. Add the information of cardiac enzyme (CK-MB).

***Response: Thank you. We have added the information of cardiac enzyme.***

1. Nitroglycerine is not appropriate to treat TC. Show the reason why authors applied this treatment.

***Response: Thank you.  We have provided the reason why nitroglycerine was used.***

1. Discussion: each paragraph should include the topics authors would like to emphasize. The mixing of short and long paragraphs makes following the right discussion points difficult.

***Response: Thank you for your feedback. This section was revised accordingly.***

1. Authors just addressed the knowledge which is already known in previous reports. Readers would like to know academic aspects from this case. To improve the quality of this case report, this reviewer recommends authors to reconsider the concept of this case.

***Response: Thank you. We have provided more information on academic aspects from the case. The message of this case was reevaluated, and it was reinforced in the conclusion.***

1. Also, I recommend the manuscript be proofread.

***Response: Thank you. The manuscript was proofread as suggested.***

# Reviewer report: Reviewer response for version 1

**DOI:** 10.5256/f1000research.134270.r144264

**Published:** 2022-07-18

**Authors:** John D. Horowitz

**Affiliations:** 1 Freemasons Centre for Male Health and Wellbeing, South Australian Health and Medical Research Institute, School of Medicine, The University of Adelaide, Adelaide, SA, Australia

This is a case report of a patient whose takotsubo syndrome (TTS: note the name) was diagnosed after an intra-abdominal operation on an elderly woman. There is a great deal wrong with the report, and I will try to cover some of it:-

1. It is not mentioned what were the findings at laparotomy. Was there, in fact, any intra-abdominal pathology?? If so, this is secondary TTS: if not, it is likely that the abdominal pain reflected the onset of TTS of primary type.
2. The clinical details are deficient. It is not mentioned specifically, but I presume that the patient was seriously hypotensive. This occurs mainly because in the acute stages of TTS, there is inappropriate vasodilatation and extravasation of fluid from the vasculature to the interstitium. This was not mentioned. The patient was treated with pressor agents, despite the fact that TTS is caused by an aberrant response to released catecholamines, and by infused nitroglycerine, despite the fact that there is already increased tissue sensitivity to nitric oxide, together with a propensity towards nitrosative stress. The correct consideration would have been fluid administration as primary care: this would have averted renal functional deterioration.
3.  TTS is neither a cardiomyopathy nor a transient disorder. It is inflammatory within the myocardium, transiting to energetic impairment, and eventually variable fibrosis. This was entirely omitted. The long-term prognosis is none too good either, partially because of an association with cancer. None of this was stated.
4.  NT-proBNP is not a natriuretic peptide: it is actually inert.35,000 represents the upper limit of most commercial assays: I suspect that the notation should be > 35,000.
5.  The case is particularly relevant because it should have stimulated discussion on the nature of peri-operative myocardial injury in general: as the authors should know, this area remains controversial and tainted by (admitted) data falsification. There is increasing evidence that many of these cases are TTS rather than infarction. None of this was mentioned.

Are enough details provided of any physical examination and diagnostic tests, treatment given and outcomes?

No

Is the case presented with sufficient detail to be useful for other practitioners?

No

Is sufficient discussion included of the importance of the findings and their relevance to future understanding of disease processes, diagnosis or treatment?

No

Is the background of the case’s history and progression described in sufficient detail?

Partly

Reviewer Expertise:

NA

I confirm that I have read this submission and believe that I have an appropriate level of expertise to state that I do not consider it to be of an acceptable scientific standard, for reasons outlined above.

# Response

**Published:** 2022-11-01

**Authors:** Virgilio E Failoc-Rojas

**Affiliations:** Unidad de Investigación para la Generación y Síntesis de Evidencias en Salud, Universidad San Ignacio de Loyola, Lima, Perú, Peru

**Response to Reviewer 2**

1. It is not mentioned what were the findings at laparotomy. Was there, in fact, any intra-abdominal pathology?? If so, this is secondary TTS: if not, it is likely that the abdominal pain reflected the onset of TTS of primary type.

***Response: Thank you. Findings on laparotomy were provided. As suggested by the report, TS was assumed to have appeared after surgery.***

1. The clinical details are deficient. It is not mentioned specifically, but I presume that the patient was seriously hypotensive. This occurs mainly because in the acute stages of TTS, there is inappropriate vasodilatation and extravasation of fluid from the vasculature to the interstitium. This was not mentioned. The patient was treated with pressor agents, despite the fact that TTS is caused by an aberrant response to released catecholamines, and by infused nitroglycerine, despite the fact that there is already increased tissue sensitivity to nitric oxide, together with a propensity towards nitrosative stress. The correct consideration would have been fluid administration as primary care: this would have averted renal functional deterioration.

***Response: Thank you for your comment. Hypotension was mentioned in the last sentence of the first paragraph in Case Report section. We agree on the suggested management of TS, we have noted this in the Discussion.***

1. TTS is neither a cardiomyopathy nor a transient disorder. It is inflammatory within the myocardium, transiting to energetic impairment, and eventually variable fibrosis. This was entirely omitted. The long-term prognosis is none too good either, partially because of an association with cancer. None of this was stated.

***Response: Thank you. As far as we know, the etiology and pathophysiology of TS remains poorly understood to date, reason for which there is still no consensus on the terminology to date*** ***https://www.ncbi.nlm.nih.gov/pmc/articles/PMC7612566/*** ***. However, we have followed the recent publications and decided to revise the term for Takotsubo syndrome. Also, we have revised the information on the prognosis as suggested.***

1. NT-proBNP is not a natriuretic peptide: it is actually inert.35,000 represents the upper limit of most commercial assays: I suspect that the notation should be > 35,000.

***Response: Thank you. We have revised the notation.***

1. The case is particularly relevant because it should have stimulated discussion on the nature of peri-operative myocardial injury in general: as the authors should know, this area remains controversial and tainted by (admitted) data falsification. There is increasing evidence that many of these cases are TTS rather than infarction. None of this was mentioned.

***Response: Thank you. We have dedicated this comment in a part of the discussion on diagnosis.***

# Reviewer report: Reviewer response for version 1

**DOI:** 10.5256/f1000research.134270.r141232

**Published:** 2022-07-05

**Authors:** Carlos Culquichicon

**Affiliations:** 1 Fred Hutch, Seattle, WA, USA

This case report presented a patient attending the emergency room due to an initial abdominal trauma. Days after the surgery, she developed Takotsubo syndrome as a complication but reversed in the discharge.

 - Provide summarized details about the "electrocardiographic changes" in the abstract section.

 - "The diagnosis of Takotsubo syndrome with cardiogenic shock and renal failure on hemodialysis was made". It is unclear whether the Takotsubo diagnosis was made during or before hemodialysis.

 - In the 3rd paragraph of the discussion section, the authors mention that some other cardiomyopathies were ruled out. It might be good to know which diseases were ruled out and the rationale.

 - It would be great if an English-native clinical expert checks the accuracy of medical terminology.

Are enough details provided of any physical examination and diagnostic tests, treatment given and outcomes?

Yes

Is the case presented with sufficient detail to be useful for other practitioners?

Yes

Is sufficient discussion included of the importance of the findings and their relevance to future understanding of disease processes, diagnosis or treatment?

Yes

Is the background of the case’s history and progression described in sufficient detail?

Yes

Reviewer Expertise:

Epidemiological methods

I confirm that I have read this submission and believe that I have an appropriate level of expertise to confirm that it is of an acceptable scientific standard.

# Response

**Published:** 2022-11-01

**Authors:** Virgilio E Failoc-Rojas

**Affiliations:** Unidad de Investigación para la Generación y Síntesis de Evidencias en Salud, Universidad San Ignacio de Loyola, Lima, Perú, Peru

**Response to Reviewer 1**

 - Provide summarized details about the "electrocardiographic changes" in the abstract section.

***Response: Thank you. We have added the suggested information.***

 - "The diagnosis of Takotsubo syndrome with cardiogenic shock and renal failure on hemodialysis was made". It is unclear whether the Takotsubo diagnosis was made during or before hemodialysis.

***Response: Thank you. We have revised the sentence for clarity.***

 - In the 3rd paragraph of the discussion section, the authors mention that some other cardiomyopathies were ruled out. It might be good to know which diseases were ruled out and the rationale.

***Response: Thank you. We have added the requested diseases and the rationale.***

 - It would be great if an English-native clinical expert checks the accuracy of medical terminology.

***Response: Thank you. The medical terminology was revised as suggested.***
